# Supplementary material for: Mechanical-scan-free multicolor super-resolution imaging with diffractive spot array illumination
Source: Nat Commun. 2024 May 16;15:4135. doi: 10.1038/s41467-024-48482-z (PMC11099116; doi:10.1038/s41467-024-48482-z)
Supplement: Supplementary file 1 — Supplementary Information [file 41467_2024_48482_MOESM1_ESM.pdf]

Mechanical-scan-free multicolor super-resolution imaging with diffractive spot array illumination: supplementary information

Ning Xu[1], Sarah E. Bohndiek[2,3], Zexing Li[4], Cilong Zhang[1], and Qiaofeng Tan[1]*

[1] State Key Laboratory of Precision Measurement Technology and Instruments, Department of Precision Instrument, Tsinghua University, Beijing 100084, China
[2] Department of Physics, Cavendish Laboratory, University of Cambridge, JJ Thomson Avenue, Cambridge, CB3 0HE, UK
[3] Cancer Research UK Cambridge Institute, University of Cambridge, Robinson Way, Cambridge, CB2 0RE, UK
[4] Department of Pure Mathematics and Mathematical Statistics, University of Cambridge, Wilberforce Road, Cambridge, CB3 0WB, UK

*Corresponding author: tanqf@mail.tsinghua.edu.cn

Supplementary Information

**Supplementary Information 1**	Characteristic and demonstration of the MCoSM
**Supplementary Information 2**	Characteristics of phase-shift scanning
**Supplementary Information 3**	Supplementary figures
**Supplementary Information 4**	Algorithm comparison with different initial phase
**Supplementary Information 5**	Analysis of uniformity across the excitation array

1 Characteristic and demonstration of the MCoSM

In the following section, we provide a framework for analyzing Mechanical-scan-free multiColor Super-resolution Microscopy (MCoSM). We will start from the relationship between illumination system and imaging system. Then, we will extend the approach used in mathematical upper boundary and intermediate value theorem to evident the number of illumination spots can be extended to $N^2\left(N \in \mathrm{N}^*\right)$. Last, the concludes from the demonstration that the super-resolution can be obtained with the N×N spot array incidence if the efficiency of the illumination is enough during practical applications. Some discussion about the MCoSM also shown in section 1.3.

1.1 Point spread function of the MCoSM

Although the super-resolution spot arrays have been generated in the focal plane, the spot arrays should be resolved in the imaging system (Fig. S1a). Typically, it is necessary to match the illumination and imaging systems for resolving the super-resolution information of spots, i.e. the numerical aperture (NA) of the imaging system need to match the NA of the illumination system. The effective point spread function (PSF) of the MCoSM can be expressed analytically as

$$P_{\text{effe}}\left(x,y\right)=\left[P_{\text{illu}}\left(x,y\right)\cdot t\left(x,y\right)\right]\otimes P_{\text{imag}}\left(x,y\right), \tag{1}$$

where the effective PSF of MCoSM $P_{\text{effe}}\left(x,y\right)$ is given by the product of the PSFs of the illumination system $P_{\text{illu}}\left(x,y\right)$ and imaging system $P_{\text{imag}}\left(x,y\right)$, and $\otimes$ denotes convolution. For the convenience of imaging, the illumination objective and the imaging objective are shared for inverted microscopes. Hence, the imaging PSF can be described by the Airy spot, which is decided be the NA of the optical system.

Generally, the pixel size of the focal plane is restricted by the equation $\Delta x = \lambda f / N \cdot \Delta p$, where Δp is the pixel size of the SLM, λ is the incident wavelength, f is the focal length of the objective, N is the sampling number of the focal plane, and N and Δp are equal to 2048 and 8 μm in the MCoSM, respectively. The distance between the central zeroth order beam and spot array center is $z = 200 \times \Delta x$. The angle of the spot array laterally offset relative to the axes of the objective is $\theta = \arctan(z / f) = \arctan(200\lambda / N \cdot \Delta p)$.

When the incident wavelength λ_1 is equal to 632.8 nm, θ_1 is equal to 0.44°; when the incident wavelength λ_2 is equal to 405 nm, θ_2 is equal to 0.28°. We believe the lateral offset of the spot array is not affected by the MCoSM, which is consistent with experimental results. The spot array is away from the spatial zeroth order to ensure the high-quality illumination (Fig. S1b).

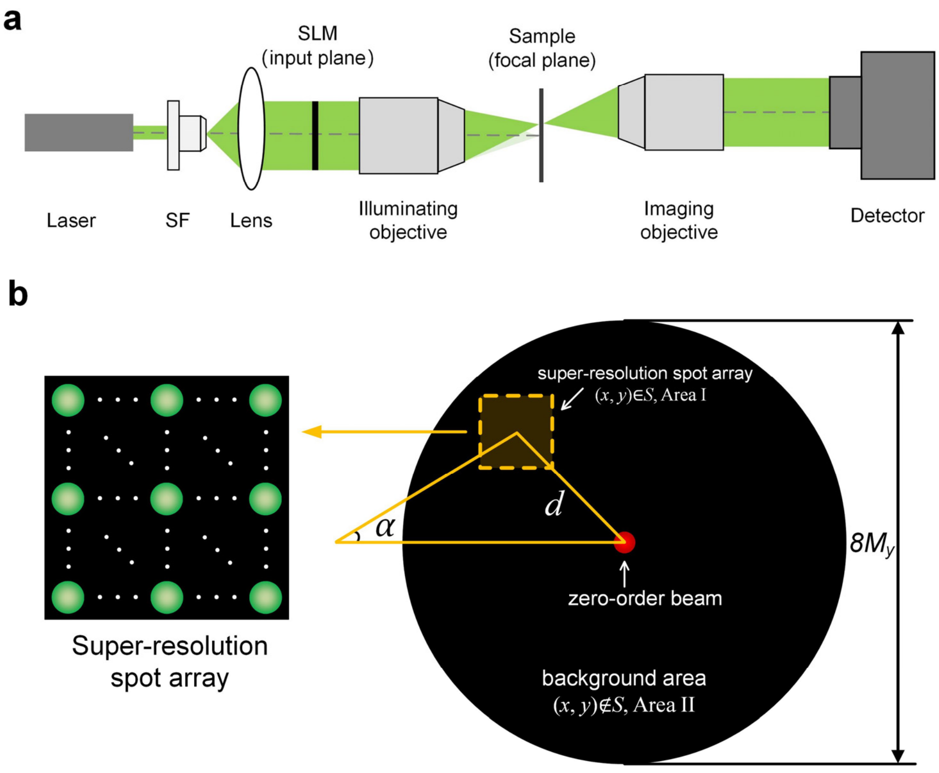

Fig. S1. (a) The transmission microscope model and (b) the spot distribution in the focal plane. Phase-only hologram distribution is uploaded on the SLM, which is inserted on the illumination part. Typically, the illumination objective and the imaging objective are shared for inverted microscopes.

1.2 Analysis of the relationship between the illumination system and imaging system

The effective PSF of MCoSM can be expressed as Eq. (1).We assume that the NAs of the imaging system and illumination system are the same (e.g. inverted microscope), the image obtained by the sensor, as shown in Fig. S2c, is the convolution of Figs. S2a and S2b. Figure S2e is the convolution of Figs. S2d and S2b, showing the flexible ability of the MCoSM. The separated spot arrays will be blurred after convolution with the imaging system, but it is clear from Figs. S2c and S2e that the relative positions and intensity values of the spot centers will not be affected by convolution because the distance between the adjacent spot centers is twice the size of the super-resolution spot in our model. The NA of the imaging objective equals that of the illumination objective, and as a consequence the super-resolution information can be obtained by the imaging system.

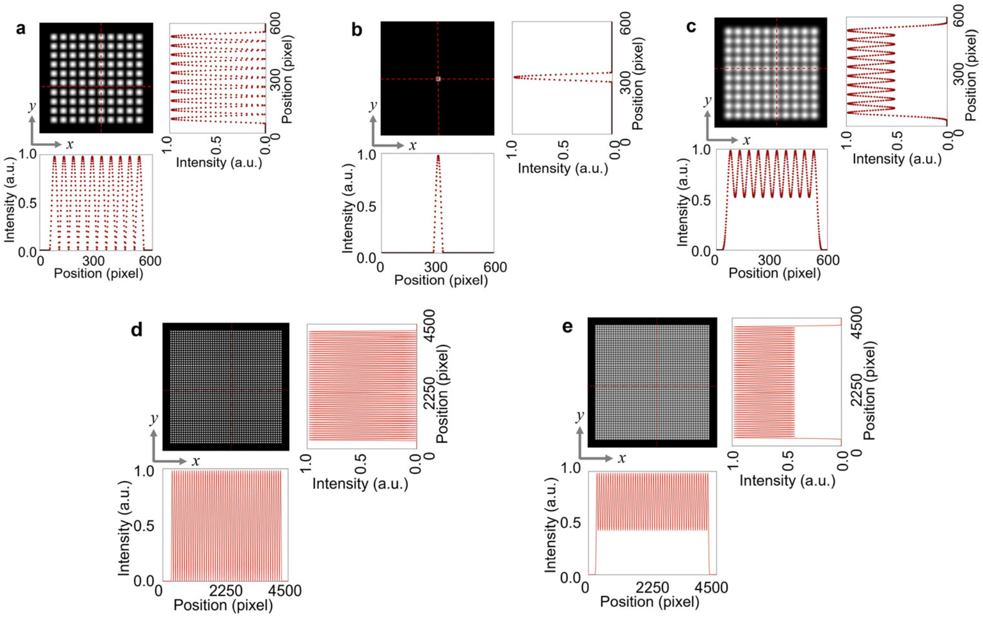

Fig. S2. Convolution analysis of super-resolution spot array illumination and imaging system. (a) 10×10 (N=10) spot array with 0.5 Airy spot size (image size: 600×600 pixels) and **(c)** 50×50 (N=50) spot arrays with 0.7 Airy spot size (image size: 4500×4500 pixels). **(b)** Airy spot with an image size of 600×600 pixels. **(e)** is the convolution of **(d)** and **(b)** with an image size of 4500×4500 pixels.

Taking a single super-resolution spot with a resolution of 0.5 Airy as an example (the red solid line in Fig. S3). The single super-resolution spot convolution with the imaging system (the blue dotted line in Fig. S3), the intensity at the detection point becomes slightly widen (the orange solid line in Fig. S3). Hence, the convolution result shows that the performance of a single super-resolution spot is limited by the diffraction limit of the imaging system, but the central position and intensity have not changed. Therefore, the central position and intensity of the obtained spot has not changed even though the illumination spot at the detection point is restricted by the imaging system after convolution and the obtained spot is not super-resolved in a single super-resolution (N=1). Similarly, the obtained intensity of the central spot can also be extracted to obtain the super-resolution information of the sample. It should

be noted that Fig. S3 does not consider the influence of the sidelobes of the super-resolution spot and other orders of Airy spots after convolution.

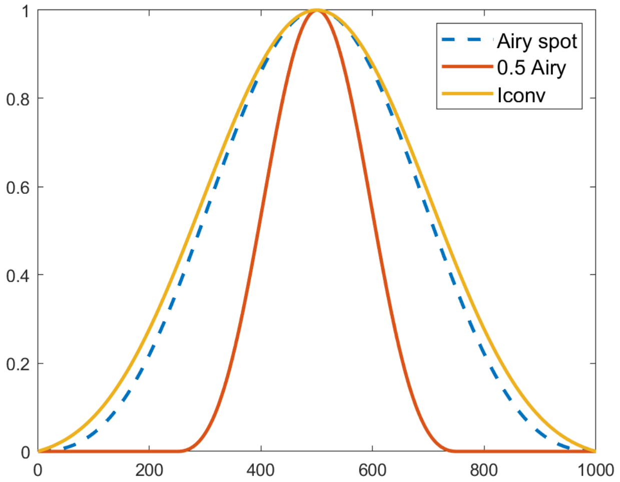

Fig. S3. Convolution analysis of single super-resolution spot illumination and imaging system. Airy spot: 1000×1 pixels, single super-resolution spot: 500×1 pixels.

By reducing the size of the illumination spots matrix and increasing the NA of the imaging system, the overlapping area of the spot array becomes smaller after imaging with separated super-resolution spot array. However, the determination of the improvement of resolution is based on the imaging objective. The best match is the NA of the imaging objective and the illumination objective are the same. Therefore, an inverted microscope shared by the illumination/imaging objective can be used to construct a super-resolution microscopic imaging system, and the super-resolution spot array needs to be sparse.

1.3 MCoSM mathematical demonstration: *N*×*N* super-resolution spot array illumination enables super-resolution imaging

In this paper, we experimentally demonstrate the multicolor super-resolution imaging through 100s-10,000s super-resolution spot illumination with different

effective field of view (FoV) imaging. In sections 1.1 and 1.2, we have analyzed the relationship between the illumination system and imaging system, and the numerical simulation verifies that the super-resolution imaging can be realized under the conditions of single super-resolution spot (N=1), 10×10 (N=10), and 50×50 (N=50) spot array illumination. We wondered whether the process for N×N super-resolution spot arrays was the same as that for 3×3 or 5×5 spot arrays after the convolution. Moreover, we wondered whether the position and intensity of the center spot (after normalization) remained constant. Here, we will prove and analyze whether the super-resolution information after convolution can be obtained when the number of spots extend to N^2 ($N \geq 1$).

Assume the NA of the illumination system is equal to the imaging system while the distance between the adjacent spot centers is twice the spot size, we mathematical demonstrate the relative positions and intensities of the N×N illumination spot array are not affected by the convolution of the imaging system. With the number of N^2 increases, it is necessary to explore whether the relative positions and intensities of the illumination spot array still unchanged. Based on this, we will demonstrate this proposition in one-dimensional N×N spot array. The proof is divided into three parts. The first part is to analyze the properties of Bessel function, which will analyze and define the properties of the subsequent Bessel function. The second part is the statement of proposition, which converts the analysis of the convolution relation of system into a mathematical proposition for proof, and expounds the preconditions for its establishment. The third part is to prove the proposition by upper bound value and intermediate value theorem.

1.3.1 The properties of J_0

The properties of the first kind of zero-order Bessel function J_0 are analyzed,

$$J_0(x) = \frac{1}{2\pi}\int_{-\pi}^{\pi} e^{-ix\sin\tau}\, d\tau, \tag{2}$$

be the Bessel function of the first kind of order zero. Define the first positive zero of as J_0, J_0'' as a , b respectively. Their values are roughly

$$a \simeq 2.4, \quad b \simeq 1.8. \tag{3}$$

We will exploit the following properties of J_0 on $[-a, a]$:

(1) *Symmetry:* J_0 is an even function. Thus J_0' is odd and J_0'' is even.

(2) *Monotonicity:* J_0'' is strictly increasing on $[0, a]$. Hence, $J_0'' < 0$ on $[0, b)$ and $J_0'' > 0$ on $(b, a]$.

(3) *Asymmetric property near b:* The following estimate holds:

$$J_0'(b+t) + J_0''(b-t) < 0, \quad \forall t \in (0, a-b). \tag{4}$$

Notations: L^1 and L^∞ norms of a function:

$$\|f\|_{L^1([x_1, x_2])} := \int_{x_1}^{x_2} |f(x)|\, dx, \tag{5}$$

$$\|f\|_{L^\infty([x_1, x_2])} := \sup_{x \in [x_1, x_2]} |f(x)|, \tag{6}$$

We use $[x]$ to denote the largest integer not larger than x, namely

$$[x] := n, \quad \text{if } n \le x \le n+1 \text{ for some interger } n. \tag{7}$$

We write $\mathbb{Z}$ for the set of integers.

Define the truncated Bessel function (Fig. S4) as

$$B(x) := J_0(x)\mathbb{I}_{[-a, a]}(x). \tag{8}$$

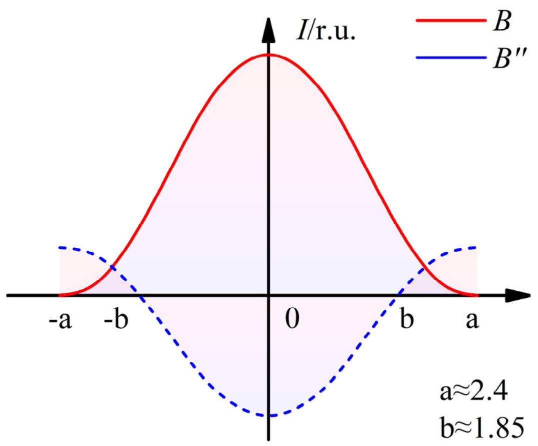

Fig. S4. Diagram of the Bessel function and its second derivative distribution, where B is the Bessel function, B'' is the second derivative of the Bessel function. $\pm a$ and $\pm b$ are the values of the intersections of the function B and B'' with the x-axis, respectively.

We can compute Eqs. (9) and (10),

$$B''(2b-a) \simeq -0.2, \tag{9}$$

$$B'(a) \simeq 0.5, \tag{10}$$

from the second derivative of Eq. (8). We can state and prove our main proposition.

1.3.2 Statement of proposition

Proposition 0.1. Let $f:\mathbb{R} \to [0,1]$ to be continuous, even and periodic with period

$$0 \le T \le \frac{4b-2a}{3}, \tag{11}$$

This is to make $\left[(4b-2a)/T\right]-2>0$, so that Eq. (13) could possibly be satisfied. Assume

$$f(kT)=1,\ f\left[\left(k+\frac{1}{2}\right)T\right]=0,\ \ \forall k \in \mathbb{Z}. \tag{12}$$

Then for every $0<\varepsilon<(1/2T)$, combining with Eq. (11), if

$$\Delta_\varepsilon := B''(2b-a)\|f\|_{L^1([0,\,T])}\left(\left[\frac{4b-2a}{T}\right]-2\right)-2B'(a)\|f\|_{L^\infty([a-\varepsilon T,\,a+\varepsilon T])}<0. \tag{13}$$

Therefore, the condition Eq. (13) roughly indicates that

$$a \simeq \left(k+\frac{1}{2}\right)T,\ \exists k\in\mathbb{Z}, \tag{14}$$

Practically, we can bound the L^1 norm of $f(x)$ by its L^1 norm on one period and the number of periods contained in $[-a,a]$:

$$\|f\|_{L^1([-a,a])}\le\|f\|_{L^1([0,T])}\left(\left[\frac{a}{T}\right]+2\right). \tag{15}$$

Combining with Eq. (13) to Eq. (15), and a function $t:\mathbb{R}\to\mathbb{R}$ satisfies

$$\|t-1\|_{L^\infty(\mathbb{R})}<\min\left\{\frac{\varepsilon T|\Delta_\varepsilon|}{\|B'\|_{L^\infty([-a,a])}\|f\|_{L^1([-a,a])}},\frac{|\Delta_\varepsilon|}{\|B'\|_{L^\infty([-a,a])}\|f\|_{L^1([-a,a])}+2|B'(a)|}\right\}, \tag{16}$$

then

(1) $B\otimes f$ takes its local maximal at $x=kT$ for $k\in\mathbb{Z}$,

(2) $B\otimes(t\cdot f)$ has a unique maximal point in $((k-\varepsilon)T,(k+\varepsilon)T)$ for every $k\in\mathbb{Z}$.

1.3.3 Proof proposition

1.3.3.1 Preparation. Firstly, we have the following formula from Eq. (1) with a general continuous function p_{illu}.

$$p_{\text{imag}}\otimes p_{\text{illu}}(x)=\int_{-a}^{a}p_{\text{imag}}(y)\,p_{\text{illu}}(x-y)\,dy$$

$$=\int_{-a}^{0} p_{\text{imag}}(y)\, p_{\text{illu}}(x-y)\,dy+\int_{0}^{a} p_{\text{imag}}(y)\, p_{\text{illu}}(x-y)\,dy$$

$$=\int_{0}^{a} p_{\text{imag}}(y)\left[p_{\text{illu}}(x-y)+p_{\text{illu}}(x+y)\right]dy. \tag{17}$$

For a symmetry optical system, the PSF of the imaging system p_{imag} can be described by $B(x)$ in the interval of $[-a,a]$.

According to the symmetry property of Bessel function. The derivative of function $p_{\text{illu}} \otimes B$ in Eq. (17) can be written as

$$(p_{\text{illu}} \otimes B)'(x)=\int_{-a}^{a} B(y) p_{\text{illu}}'(x-y)\,dy=-\int_{-a}^{a} B(y)\,\partial_y p_{\text{illu}}(x-y)\,dy$$

$$=-\left[B(y)\, p_{\text{illu}}(x-y)\right]\Big|_{y=-a}^{a}+\int_{-a}^{a} \partial_y B(y)\, p_{\text{illu}}(x-y)\,dy \tag{18}$$

$$=\int_{0}^{a} B'(y)\left[p_{\text{illu}}(x-y)-p_{\text{illu}}(x+y)\right]dy. \tag{19}$$

Notably, in the last equivalence of Eqs. (18) to (19), we used $B(a)=B(-a)$ to make the boundary term vanish and the oddness of $B'(x)$. Similarly, via integration by parts, the second order derivative of $p_{\text{illu}} \otimes B$ is

$$(p_{\text{illu}} \otimes B)''(x)=\int_{-a}^{a} B(y) p_{\text{illu}}''(x-y)\,dy=\int_{-a}^{a} B'(y)\, p_{\text{illu}}'(x-y)\,dy$$

$$=\int_{-a}^{a} B''(y) p_{\text{illu}}(x-y)\,dy-B'(a)\, p_{\text{illu}}'(x-a)+B'(-a)\, p_{\text{illu}}'(x+a)$$

$$=\int_{-a}^{a} B''(y) p_{\text{illu}}(x-y)\,dy-B'(a)\left[p_{\text{illu}}(x-a)+p_{\text{illu}}(x+a)\right]. \tag{20}$$

We will apply these formulas for $p_{\text{illu}}=f$ and $p_{\text{illu}}=(t-1)f$.

From the symmetry of Bessel function and the symmetry of *N*×*N* spot array, Eq. (19) can be expressed as

$$(p_{\text{illu}} \otimes B)'(kT)=0,\ \forall k \in \mathbb{Z}. \tag{21}$$

where *T* is the period of spot array (Fig. S5).

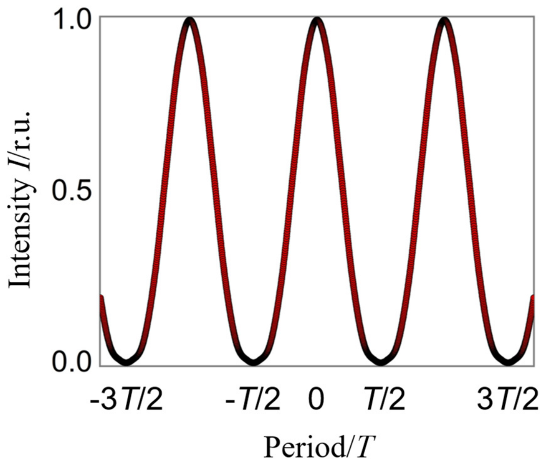

Fig. S5. Super-resolution spot array (one-dimensional) with period *T*.

From f is even and T-periodic, for $\forall k \in \mathbb{Z}$, $\forall y \in \mathbb{R}$, we can easily derive

$$f\left(\frac{1}{2}kT + y\right) = f\left(\frac{1}{2}kT - y\right). \tag{22}$$

For $\forall k \in \mathbb{Z}$, Eqs. (19) and (21) indicate

$$\left(B \otimes f\right)'\left(kT\right)=0. \tag{23}$$

1.3.3.2 Upper bound of function $\left(B \otimes f\right)''$

Next, we show the Δ_ε is an upper bound for near 0. More precisely, we can transform the question into proof Eq. (23), and we will show

$$\Delta_\varepsilon \geq \sup_{x \in [-\varepsilon T,\ \varepsilon T]} \left(B \otimes f\right)''\left(x\right). \tag{24}$$

Since $B'\left(a\right) \simeq -0.5 < 0$, for $\forall \left|x\right| \leq \varepsilon T$, the second part in Δ_ε (in Eq. 13) clearly bounds the second term in Eq. (20)

$$-B'\left(a\right)\left[f\left(x-a\right)+f\left(x+a\right)\right] \leq -2B'\left(a\right)\left\|f\right\|_{L^\infty\left(\left[a-\varepsilon T,\ a+\varepsilon T\right]\right)}. \tag{25}$$

For the first term in Eq. (19), the main contribution is

$$\int_{-(2b-a-T)}^{2b-a-T} B''\left(y\right)f\left(x-y\right)dy \leq B''\left(2b-a\right)\left\|f\right\|_{L^1\left([0,\ T]\right)}\left(\left[\frac{4b-2a}{T}\right]-2\right).$$

(26)

where we used the monotonicity of B''. Finally, we prove the following cancellation estimates, for any $x \in \mathbb{R}$, to proof Eq. (27) and Eq. (28)

$$\int_{2b-a-T}^{a} B''(y) f(x-y)\,dy \le 0, \tag{27}$$

$$\int_{-a}^{-(2b-a-T)} B''(y) f(x-y)\,dy = 0. \tag{28}$$

We only prove the first Eq. (27) and the second Eq. (28) follows similarly. There exists $k_0 \in \mathbb{Z}$ such that $\frac{1}{2}k_0 T \in \left(x-b, x-b+\frac{1}{2}T\right]$. Denote

$$\delta := \frac{1}{2}k_0 T - x + b \in \left[0, \frac{1}{2}T\right). \tag{29}$$

Then Eq. (29) can be expressed as

$$\begin{aligned}
&\int_{2b-a-T}^{a} B''(y) f(x-y)\,dy \\
&\le \int_{b}^{a} B''(y) f(x-y)\,dy + \int_{2b-a-2\delta}^{b-2\delta} B''(y) f(x-y)\,dy \\
&= \int_{b}^{a} B''(y) f\left(\frac{1}{2}k_0 T + b - \delta - y\right) dy \\
&\qquad + \int_{2b-a-2\delta}^{b-2\delta} B''(y) f\left(\frac{1}{2}k_0 T + b - \delta - y\right) dy \\
&= \int_{\delta}^{\delta+a-b} B''\left[b + (z - f)\right] f\left(\frac{1}{2}k_0 T - z\right) dz \\
&\qquad + \int_{\delta}^{\delta+a-b} B''\left[b - (z + \delta)\right] f\left(\frac{1}{2}k_0 T + z\right) dz \\
&\le \int_{\delta}^{\delta+a-b} \left\{B''\left[b + (z - \delta)\right] + B''\left[b - (z - \delta)\right]\right\} f\left(\frac{1}{2}k_0 T + z\right) dz
\end{aligned}$$

. (30)

$$\le 0, \tag{31}$$

where in the second to last inequality, we applied the symmetry of f (by Eq. (22)) and monotonicity of B'', and the last inequality follows Eq. (4). Summing up Eqs. (25) to (28), we obtain Eq. (24).

1.3.3.3 Concluding the proof

Now we conclude the proof with Eqs. (13) and (16). Noticing that $B\otimes f$ is also periodic, we only focusing on maximal property near $x=0$ for simplicity. The statement (1) follows directly Eq. (23) and

$$(B\otimes f)''(0)\leq \Delta_\varepsilon < 0. \tag{32}$$

Moreover, we have estimate of the variation of derivative of $B\otimes f$ near $x=0$

$$(B\otimes f)'(\varepsilon T)=(B\otimes f)'(0)+\int_0^{\varepsilon T}(B\otimes f)''(y)\,dy \leq \varepsilon T\Delta_\varepsilon, \tag{33}$$

$$(B\otimes f)'(-\varepsilon T)=(B\otimes f)'(0)-\int_{-\varepsilon T}^{0}(B\otimes f)''(y)\,dy \geq -\varepsilon T\Delta_\varepsilon. \tag{34}$$

Besides, we have the trivial upper bounds related to $B\otimes\left[(t-1)f\right]$ using Eq. (19) and Eq. (20):

$$\left|\left\{B\otimes\left[(t-1)f\right]\right\}'(x)\right| \leq \|B'\|_{L^\infty([-a,a])}\|f\|_{L^1([-a,a])}\|t-1\|_{L^\infty(\mathbb{R})}, \tag{35}$$

$$\left|\left\{B\otimes\left[(t-1)f\right]\right\}''(x)\right| \leq \left[\|B''\|_{L^\infty([-a,a])}\|f\|_{L^1([-a,a])}+2\left|B'(a)\right|\right]\cdot\|t-1\|_{L^\infty}. \tag{36}$$

Combining with Eq. (16), Eq. (35) and Eq. (36) can be written as

$$\left\|\left\{B\otimes\left[(t-1)f\right]\right\}'\right\|_{L^\infty} < \varepsilon T\left|\Delta_\varepsilon\right|, \tag{37}$$

$$\left\|\left\{B\otimes\left[(t-1)f\right]\right\}''\right\|_{L^\infty} < \left|\Delta_\varepsilon\right|. \tag{38}$$

Consequently, combining with Eq. (32), for any $\forall x\in[-\varepsilon T,\varepsilon T]$, we see

$$\left[B\otimes(t\cdot f)\right]'(\varepsilon T) < 0, \tag{39}$$

$$\left[B\otimes\left(t\cdot f\right)\right]'\left(-\varepsilon T\right) > 0, \tag{40}$$

$$\left[B\otimes\left(f\cdot T\right)\right]''\left(x\right) < 0. \tag{41}$$

By intermediate value theorem [1], there is one unique $x^{*}\in\left[-\varepsilon T,\varepsilon T\right]$ such that $\left[B\otimes\left(t\cdot f\right)\right]'\left(x^{*}\right)=0$ and we also know $\left[B\otimes\left(t\cdot f\right)\right]''\left(x^{*}\right)<0$. Therefore, x^{*} is the unique maximal point of $B\otimes\left(t\cdot f\right)$ near $x=0$.

1.4 Discussion and conclusion

Through 100-2500 super-resolution spots illumination with different effective FoV imaging, we demonstrate the adjustable capacity of MCoSM. To explore whether the super-resolution ability of MCoSM will be affected with the increase of the number of illumination spots, we demonstrated and analyzed with N^2 spots by upper boundary and intermediate value theorem.

From sections 1.1, 1.2 and 1.3, we conclude that, for N×N super-resolution spot array, the relative positions and intensities of the illumination spot array still unchanged after convolution with the imaging system if the sample is a thin sample, i.e., the transmittance function of the sample is satisfied with $0\le t\le\left|\Delta_{\varepsilon}\right|$ for the dark-background with bright sample or $0\le 1-t\le\left|\Delta_{\varepsilon}\right|$ for the bright-background with dark sample. In addition, the resolution of the super-resolution spots can be high enough (the size can be small enough like δ function). Although the number of spots can be large enough, the relative positions and intensities of the illumination spot array still unchanged. Notably, considering the effects of sidelobes and speckles during diffractive optical element design, we can only acquire the illumination of 50×50 spot arrays with spot sizes of 0.68 Airy by proposed algorithm nowadays. It is possible to achieve even higher resolutions by improving the optimization algorithm to obtain better super-resolution enhancement in the future.

2 Characteristics of phase-shift scanning

In this section, we provide a framework about phase-shift scanning theory and present the detailed derivation of the scanning step size. We will start from the derivation of phase-shift scanning where we want to find the relationship between the MCoSM parameters and step size. Next, we will show the characteristics with/without adding the phase of minimum scanning step.

2.1 Derivation of phase-shift scanning

In MCoSM, we describe the step of phase-shift scanning that quantitatively link the SLM and objective parameters in spatial domain. The phase of SLM from 0 to 255 can be regarded as a multi-step blazed grating. Assuming that the blazed grating is periodically arranged by *N* small prisms (Fig. S6a), where α is the apex angle of the prism, and n is the refractive index.

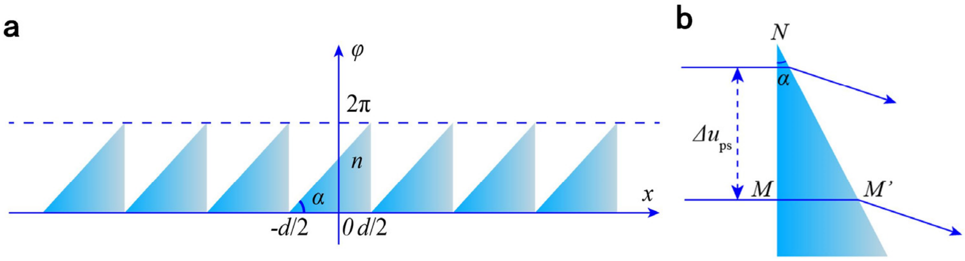

Fig. S6. The schematic of blazed grating with plane wave incidence. a Blazed grating is periodically arranged by *N* small prisms. **b** A prism with the apex angle α.

For a single prism (Fig. S6b), the optical path difference is equal to $(n-1)\overline{MM'}=(n-1)\alpha x_0$, the corresponding phase difference is $k(n-1)\alpha x_0$, and the phase difference for a single prism can be expressed as [2]

$$t_0(x)=\exp\left[jk(n-1)\alpha x\right]\mathrm{rect}\left(\frac{x}{d}\right). \quad (42)$$

where x-plane is the input plane and $k=2\pi/\lambda$ .Thus, the transmittance function of the blazed grating can be expressed as

$$t(x)=\left[\exp\left(\mathrm{j}\frac{2\pi}{d}x+\pi\right)\mathrm{rect}\left(\frac{x}{d}\right)\right]\otimes\sum_{m=-\infty}^{+\infty}\delta(x-md)\,. \tag{43}$$

The complex field distribution, u-plane in the focal plane, can be calculated by Fraunhofer diffraction

$$\begin{aligned}U(u)&=\mathcal{F}\left[t(x)\right]=\mathcal{F}\left[\exp\left(\mathrm{j}\frac{2\pi}{d}x+\pi\right)\mathrm{rect}\left(\frac{x}{d}\right)\right]\mathcal{F}\left[\sum_{m=-\infty}^{+\infty}\delta(x-md)\right],\\&=-\int_{-d/2}^{d/2}\exp\left[\mathrm{j}\cdot2\pi\left(\frac{1}{d}-\frac{u}{\lambda f}x\right)\right]dx\cdot\frac{1}{d}\sum_{m=-\infty}^{+\infty}\delta\left(u-\frac{m\lambda f}{d}\right),\\&=-\sin\mathrm{c}\left[\left(\frac{1}{d}-\frac{u}{\lambda f}\right)d\right]\sum_{m=-\infty}^{+\infty}\delta\left(u-\frac{m\lambda f}{d}\right).\end{aligned} \tag{44}$$

Considering the magnification of the system, the general phase shift step Δu_{ps} can be expressed as

$$\Delta u_{\mathrm{ps}}=\frac{2\lambda f}{d}=\frac{2\lambda f}{M_{\mathrm{sys}}N\cdot\Delta p}, \tag{45}$$

where Δp is the pixel size of the SLM, M_{sys} is the magnification of the MCoSM, and f is the focal length of the objective. It indicates from Eq. (45) that the step size of phase-shift scanning is influenced by the incident wavelength. The magnification of the MCoSM, the 6:5 magnified image (f_1=150mm, f_2=125mm) generated by SLM after spatial filtering needs to be conjugated with the sample plane through a 4f system. Therefore, the scaling ratio from the spatial filter plane to the sample plane can be written as

$$M_{\mathrm{sys}}=M_{\mathrm{obj}}\frac{f_{\mathrm{lens2}}}{f_{\mathrm{lens1}}}=60\times\frac{150}{125}=50. \tag{46}$$

2.2 Characteristics of phase-shift scanning

The phase-shift scanning method depends on the tilted phase added to the SLM, the angle and number of fringe patterns, in one period, determine the phase-shift scanning step. Through adjusting duty ratio of phase distribution, the structure of duty ratio 0-π (255 level in SLM) is modified. In unit space, the smaller the duty ratio of π, the smaller angle can achieve.

Before we calculate the step of phase-shift scanning, it should be claimed that the spot array is offset relative to axes because the zero-order generated by SLM is inevitable (Fig. S7a). And we need to calculate this angle before we design the phase of phase-shift scanning. The spot array is coaxial with the imaging system when adjusting the optical path. The effective FoV of the spot array (α) can be calculated as $\alpha = \arctan(d / f) = \arctan[200\lambda / (M \cdot \Delta p)]$, where f is the focal length of the objective, M is the sampling number of the focal plane. When the incident wavelength is equal to 561 nm, α is equal to 0.39°. Hence, the laterally offset of the spot array is not affected by the MCoSM imaging while the spot array is away from the spatial zeroth order by aperture to ensure the high-quality beam.

The characteristics (period/frequency, intensity, and profile) of spot array does not change, indicating that adding the phase distribution of phase-shift scanning does not affect the quality of the spot array (Figs. S7b and S7c). Position 1 (P1) showed the result of 10×10 super-resolution spot array without adding the phase distribution of phase-shift scanning. Position 2 (P2) showed the result of 10×10 super-resolution spot array with phase-shift scanning. Position 3 (P3) presented the result of the phase-shift scanning with the focal spot.

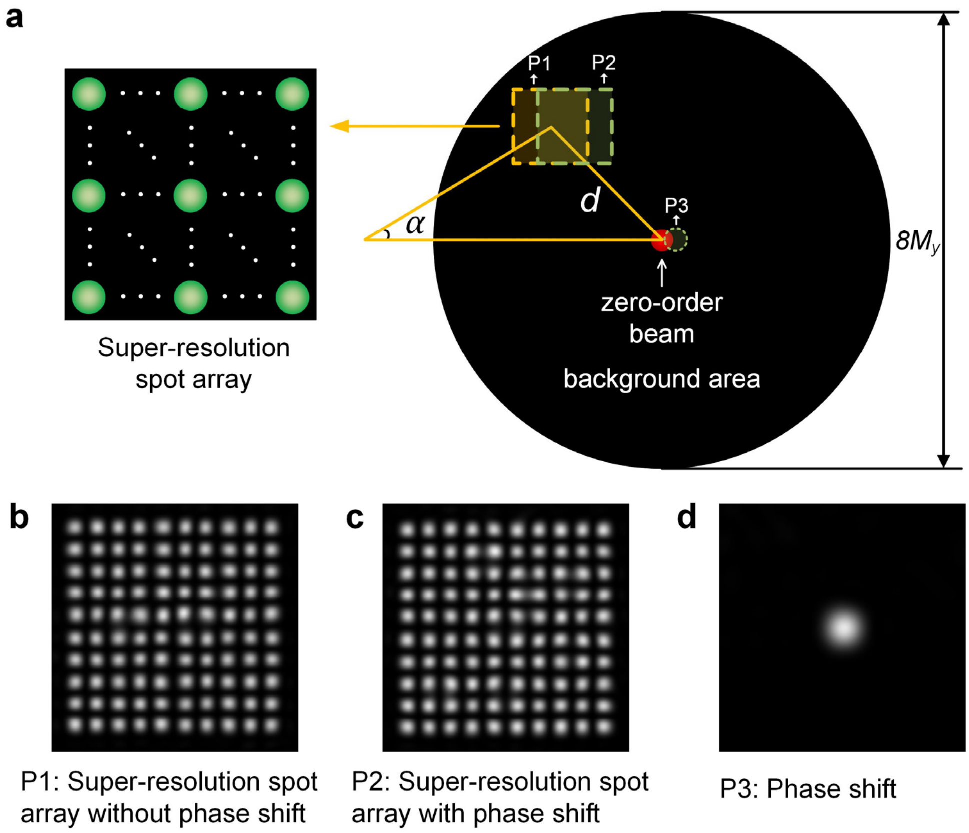

Fig. S7. Characteristics of phase-shift scanning with super-resolution spot array incidence. a The distribution of the super-resolution spot array in the focal plane. The angle of the spot array laterally offset relative to the axes of the objective is α, and the distance between the zero-order spot and spot array center is *d*. P1 and P2 are the position of the super-resolution spot array without **b** /with **c** phase-shift scanning hologram. **d** Only the distribution of the phase-shift scanning hologram is added on the SLM.

To evaluate the performance of spot array at the position of near maximum scanning range in practical applications, we experimental obtained 10×10 spot array with the spot sizes of 0.52Airy when using phase-shift scanning at the maximum position of 208 μm (Fig. S8). Due to aberration constrained of the system, we only realized the effective FoV of 167.4 μm×167.4 μm in practical

applications. In the future, we will explore the compensation of the illumination aberration to achieve a larger effective FoV and reduce the number of scans.

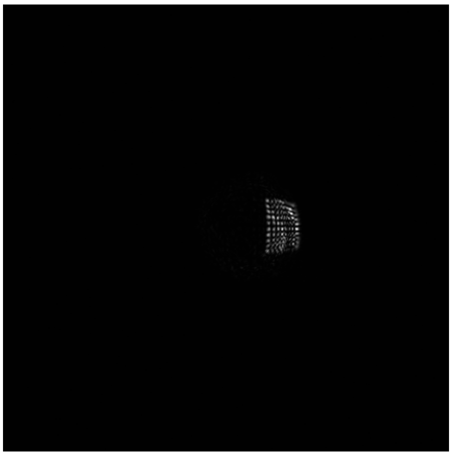

Fig. S8. Characteristics of the spot array when the scan range is near to the theoretical maximum position (208 μm). Experimental result of spot array with the NA=1.25, λ=488 nm.

3 Supplementary figures

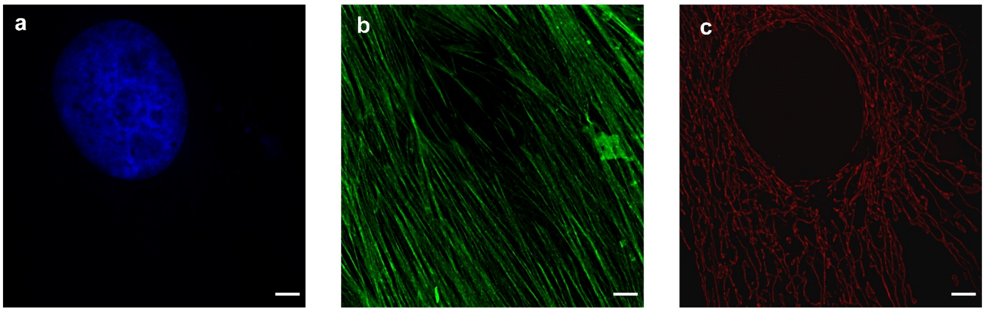

Fig. S9. The distributions of organelles over the FoV with three-colors incidence. The images at the wavelength of **a** 405 nm (nucleus), **b** 488 nm (cytoskeleton), and **c** 561 nm (mitochondria) are separately reconstructed with a negligible cross talk. Scale bar: 5 μm.

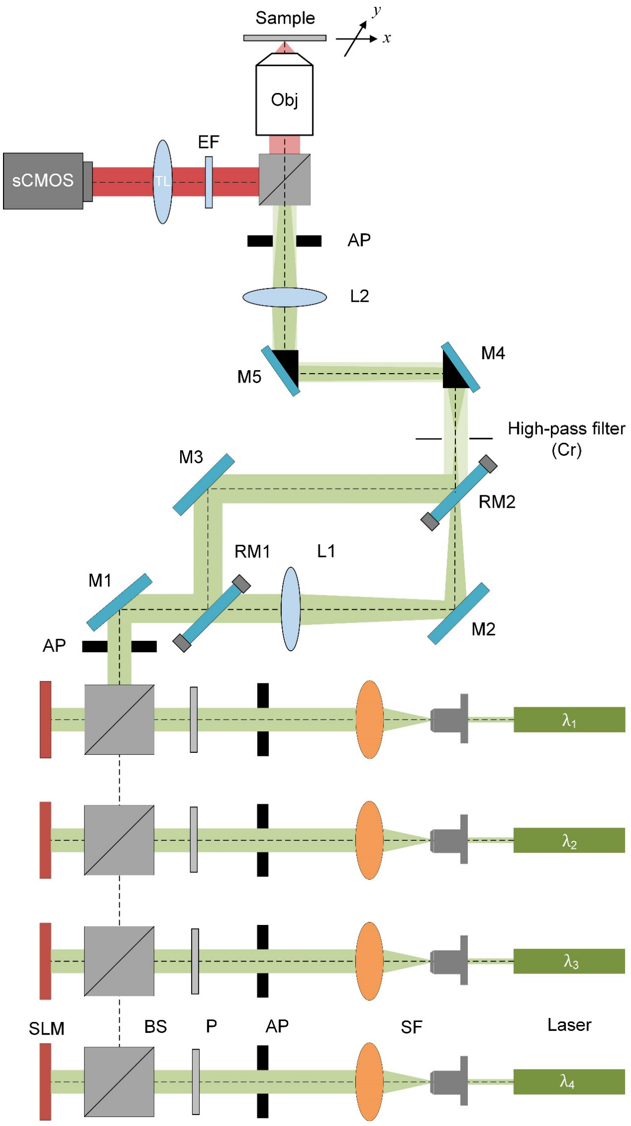

Fig. S10. Experimental setup of the MCoSM. The experimental setup consists of two main system: illumination system and imaging system. A collimated laser beam illuminates the SLM via a beam splitter. The designed phases uploaded on the SLMs are diffracted in the sample through a microscope objective. Sample scanned by phase-shift scanning was captured onto the sCMOS through tube lens and emission filter. Removable mirrors (RMs) 1 and 2 were assisted by a removable bracket using an indexing mount, which was placed to determine the position of the sample using widefield illumination. SF, spatial filter; AP, aperture; P, polarizer; BS, beam splitter; SLM, spatial light modulator; M, mirror; RM, removable mirror; L, lens; Obj, objective; EF, emission filter; TL, tube lens.

4 Algorithm comparisons with different initial phase

To validate the feasibility of proposed algorithm, numerical simulations are performed for quantitative evaluation. The performance of the proposed method is compared with the iterative Fourier transform algorithm (IFTA) [3]. To compare the algorithms, root-mean-square error (RMSE) and nonuniformity are used to compare the generated super-resolution spot array and ideal super-resolution spot array. The RMSE is calculated as

$$\mathrm{RMSE}=\sqrt{\sum_{m=1}^{M}\sum_{n=1}^{N}\left[I_g(m,n)-I_i(m,n)\right]^2/\sum_{m=1}^{M}\sum_{n=1}^{N}\left[I_i(m,n)\right]^2}\ , \tag{47}$$

where M and N are the horizontal and vertical resolution of images, $I_g(m,n)$ is the intensity distribution of the generated super-resolution spot array, and $I_i(m,n)$ is the intensity distribution of the ideal super-resolution spot array. The RMSE indicates how well the generated super-resolution spot array agrees with the target one. The lower the value of RMSE is, the better the generation quality will be. The nonuniformity of the generated spot array profile can be given by

$$U_g=\sqrt{\sum_{m=1}^{M}\sum_{n=1}^{N}\left[\frac{I_g(m,n)-\bar{I}}{\bar{I}}\right]^2/(n-1)}\ , \tag{48}$$

where $\bar{I}$ is the average intensity distribution of the generated super-resolution spot array. The value of U_g varies from 0 to 1, and smaller U_g indicates better uniformity.

In the comparisons of the IFTA and our proposed method, the ideal spot array is set as 10×10 with a resolution of 50% of the Airy spot size, the number of iterations is set as 120. The RMSE and U_g calculated by Eqs. (47) and (48) are plotted in Figs. S11a and S11b, respectively.

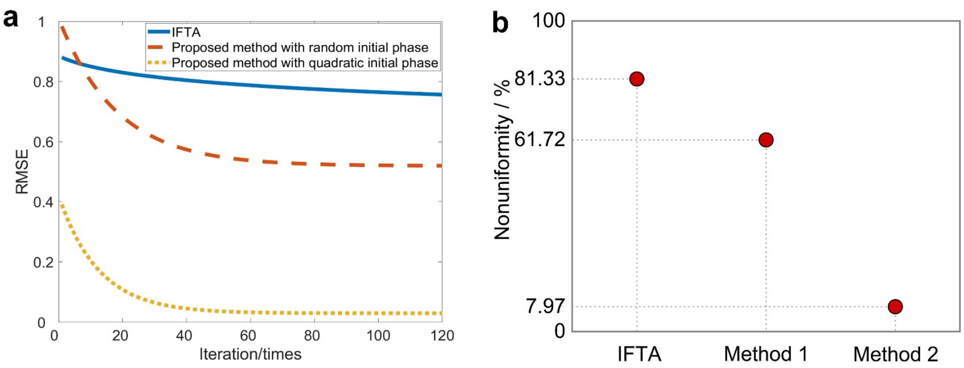

Fig. S11. Numerical simulation of different algorithms. a Comparison of the root-mean-square errors (RMSE) with IFTA algorithm, proposed method with random initial phase, and proposed method with quadratic initial phase. **b** Comparison of the nonuniformity of the spot array by different method, where method 1 and 2 are the proposed method with random initial phase and quadratic initial phase, respectively.

5 Analysis of uniformity across the excitation array

In the mathematical demonstration part, we confirmed that the relative positions and intensities of the *N*×*N* illumination spot array are not affected by the convolution of the imaging system. However, this verification presupposes standard spot sizes, shapes and intensities. During the experimentation, efforts to enhance the uniformity of the spot array were made, yet complete uniformity was unattainable due the small spot size, as depicted in Fig. 2. Non-uniformity leads to alterations in relative positions and intensities upon convolution by the imaging system. Here, we investigated the impact of uniformity across the excitation array in Fig. 2(a), featuring 10×10 spot array with a spot size of 0.52 Airy spot units.

Assume the intensity and position of the i_{th} spot as I_i and x_i, respectively. After convolution, the intensity and position change to I_{ci} and x_{ci}. We assessed the intensity ratio I_{ci}/I_i and position deviation represented as $\|x_{ci}-x_i\|$ for the spot array. As illustrated in Fig. S12, the intensity ratio deviation remains among ±15% and the position deviation is less than 15 nm. Overall, given the resolution of our imaging system, such deviations do not significantly affect the structural information of biological samples.

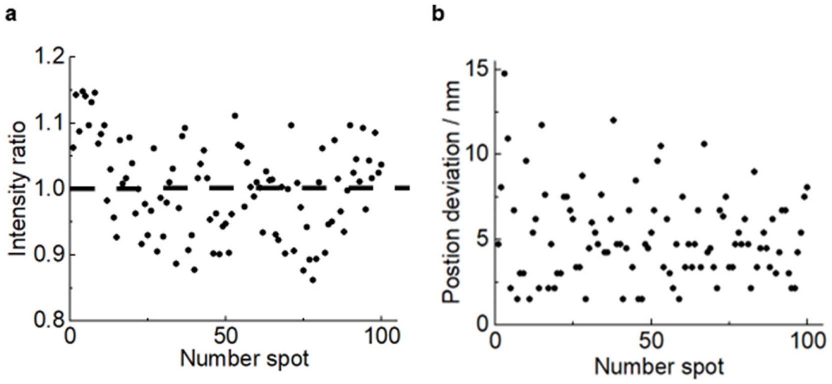

Fig. S12. Analysis of uniformity across the excitation array. The results of **a** Intensity ratio, **b** Position deviation due to the un-uniformity across the excitation array.

References

1. A. Douglas, “Essentially follows Clarke,” Foundations of analysis appleton-century-crofts, pp. 284, (1971).
2. M. Born and E. Wolf, “Principles of Optics,” Cambridge University Press, Cambridge (1999).
3. F. Wyrowski and O. Bryngdahl, “Iterative Fourier-transform algorithm applied to computer holography,” J. Opt. Soc. Am. A **5**, 1058-1065 (1988).
