# Supplementary material for: Mechanical-scan-free multicolor super-resolution imaging with diffractive spot array illumination
Source: Nat Commun. 2024 May 16;15:4135. doi: 10.1038/s41467-024-48482-z (PMC11099116; doi:10.1038/s41467-024-48482-z)
Supplement: Supplementary file 3 — Description of Additional Supplementary Files [file 41467_2024_48482_MOESM3_ESM.pdf]

Description of Additional Supplementary Files

Supplementary Movie 1:

Supplementary Movie 1 shows a 10×10 spot array with a resolution of 0.52 Airy on fluorescence beads with phase-shift scanning. The objective with NA equal to 1.25 and the excitation wavelength of 561 nm is used.

Supplementary Movie 2:

Supplementary Movie 2 shows a 10×10 spot array with a resolution of 0.52 Airy on nucleus of BPAE with phase-shift scanning. The objective with NA equal to 0.90 and the excitation wavelength of 405 nm is used.

Supplementary Movie 3:

Supplementary Movie 3 shows a 10×10 spot array with a resolution of 0.52 Airy on cytoskeleton of BPAE with phase-shift scanning. The objective with NA equal to 0.90 and the excitation wavelength of 488 nm is used.
